# An 8‐year retrospective study of fixed prosthodontics clinical unit completions in a postgraduate prosthodontics program

**DOI:** 10.1002/hsr2.1331

**Published:** 2023-06-12

**Authors:** James Dudley

**Affiliations:** ^1^ Adelaide Dental School The University of Adelaide Adelaide Australia

**Keywords:** clinical units, fixed prosthodontics, postgraduate prosthodontics, retrospective study

## Abstract

**Background and Aims:**

The range of aesthetic fixed prosthodontics materials utilizing digital manufacturing techniques has expanded in recent years ostensibly replacing traditional laboratory techniques and materials. This retrospective study conducted over eight consecutive years aimed to analyze the types of laboratory fabricated fixed prosthodontics clinical units completed in a postgraduate prosthodontics specialist training program and determine meaningful trends.

**Methods:**

The logbooks of eight postgraduate prosthodontics completions from 2014 to 2021 were reviewed and the different types of laboratory fabricated fixed prosthodontics units and total number of fixed prosthodontics units completed were recorded. The data was categorized and presented in tabulated and chart form using Microsoft Excel software (version 2016). Paired *t*‐tests and Mann−Kendall trend tests were performed to analyze for statistical significance between the different restoration types across the program completions.

**Results:**

Porcelain bonded to metal (PBM) crowns represented 42.05% of all fixed prosthodontics units completed over all study years followed by all‐ceramic crowns (ACC) (18.14%) and full gold crowns (FGC) (10.70%). Jointly, PBM, ACC and FGC's encompassed 70.88% of all fixed prosthodontics units. Over the 8‐year study period, there were observed trends of reduced use of PBM's, increased use of ACC's, statistically significant reduced use of FGC's (*p* = 0.035) and a statistically significant difference in the use of complete and partial coverage restorations (*p* < 0.001).

**Conclusion:**

PBM crowns were the dominant laboratory fabricated fixed prosthodontic clinical unit across postgraduate prosthodontics program completions. The trend in later years indicating ACC as the dominant crown type warrants further investigation.

## 1 INTRODUCTION

Prosthodontics is the dental specialty pertaining to the diagnosis, treatment planning, rehabilitation and maintenance of the oral function, comfort, appearance and health of patients with clinical conditions associated with missing or deficient teeth and/or maxillofacial tissues using biocompatible substitutes.^1^ The training of prosthodontists serves to supply appropriate numbers of practitioners in a niche specialty that deals with complex dental cases such as oral rehabilitations and managing refractory patients.

The postgraduate prosthodontics program at this institution comprises a 3‐year, full time training program leading to qualification as a specialist prosthodontist. The program undergoes accreditation every 5 years and aligns with the competencies expected of a graduate specialist in the discipline.^2^ The program comprises a mix of clinical treatment; didactic, clinical and case presentation seminars; and a major research project. The clinical component involves the provision of treatment for patients referred to the specialist prosthodontic unit which operates within a public sector specialist dental clinic. All patients are initially assessed by general dentists then referred to the specialist clinic according to the level of complexity of their treatment. Patients are required to be eligible for treatment in the public health care system and make financial co‐payments that varies according to the clinical procedure.

The university enrolled postgraduate students hold the title of “registrar” in reference to their conjoint employment status within the public sector health service. Throughout the program, the postgraduate registrars receive continual feedback and regular formative review of their work. Summative assessments are conducted at the end of each semester/year and involves presentation of selected clinical cases and submission of a logbook of completed cases that evidence the range of clinical cases managed with emphasis on quality and reflection rather than achieving set quotas.

Assessment of performance in postgraduate/postdoctorate dental specialist training programs is generally a process intrinsic to the university and referenced against required competencies; there are no known reports on this topic in the literature to date. In the US, Board examinations are conducted after graduation that serve to equilibrate the standard of the graduating prosthodontist, however these examinations are not compulsory to practice clinically as a specialist prosthodontist. Some reports have examined program entry processes^3^ with the proposed introduction of national qualifying exams to evaluate applicants for entering programs.^4^ Other reports have investigated students' perceptions of prosthodontics and the motivation for entering the specialty with enjoyment found to be the most important factor.^5^ However, all such information gaining processes are separate to assessments of students during their course of study.

Fixed prosthodontics is one of the five subdisciplines in the postgraduate prosthodontics program. Prosthodontists are required to remain at the forefront of clinical techniques and have a detailed knowledge of traditional, modern and unusual types of crown materials that often form a key part of specialist referrals and treatments. The use of novel digital manufacturing techniques associated with more efficient, accurate and repeatable results is fundamental to this knowledge, with such technologies recently introduced at this institution.^6^, ^7^ Although most registrars entering postgraduate prosthodontics programs have good general dentistry clinical experience, preclinical courses can to some extent introduce postgraduate registrars to specialist level subdiscipline of fixed prosthodontics. At an undergraduate level, fixed prosthodontics preclinical grades have been positively correlated with clinical grades in operative dentistry and fixed prosthodontics^8^, ^9^ however it is clinical practice that forms the core in postgraduate prosthodontics programs.

Unit completions can be used as one measure of student progress.^10^ Postgraduate prosthodontics registrars maintain a logbook that chronicles unit completions using the accepted schedule describing the different units.^11^ There is no limit on the scope of treatment and generally in this program more complex fixed prosthodontics treatment is completed for patients. The range of indirect materials available for use includes all types of ceramics; zirconia; precious, semiprecious and nonprecious alloys; and the relevant combinations of materials for example porcelain bonded to metal (PBM) crowns.

Throughout the study's duration, at this institution there has been expansion of the range of new aesthetic materials using digital manufacturing techniques ostensibly replacing traditional laboratory techniques and materials coupled with continued use of traditional laboratory techniques and materials. The use of new materials has provided more options for delivering prosthodontic care to patients.

This study aimed to retrospectively review the types of laboratory fabricated fixed prosthodontics clinical units completed in a postgraduate prosthodontics specialist training program over 8 consecutive years and determine any significant trends. The null hypotheses were:

1. There were no differences between the total numbers of fixed prosthodontics unit types for the eight postgraduate prosthodontics completions, and
2. There were no significant positive or negative trends in the use of fixed prosthodontics units over the 8 year study period.

The research hypotheses were:

1. There were differences between the total numbers of fixed prosthodontics unit types for the eight postgraduate prosthodontics completions, and
2. There were significant positive or negative trends in the use of fixed prosthodontics units over the 8 year study period.

The level of significance was set at *p* = 0.05.

## 2 METHODS

### 2.1 Data collection and analysis

The logbooks of postgraduate prosthodontics completions from 2014 to 2021 were obtained and each registrar completion was numbered in chronological order of program completion from 1 to 8. For each completion, the different types of laboratory fabricated fixed prosthodontics units and the total number of fixed prosthodontics units completed were recorded. The instructions provided to registrars for maintaining logbooks were consistent across the study period. The logbook recording was checked against clinical records and cross‐checked by a fellow academic at the time of data analysis.

The different types of fixed prosthodontics restorations were firstly categorized (Table 1) then totaled for each restoration category and expressed as a percentage of the total number of units completed for each program completion. Data was presented in tabulated and chart form using Microsoft Excel software (version 2016).

**Table 1:** Categorization of the different fixed prosthodontics restorations.

Type of restoration	Abbreviation	Types of restorations included
Porcelain bonded to metal crown	PBM	All types of PBM's including metal occlusal designs
Full gold crown	FGC	All FGC's not involving any veneering material, including all metal alloy types
All‐ceramic crown	ACC	E.max (IPS e.max, Ivoclar Vivadent), porcelain‐bonded to zirconia and full contour zirconia crowns
Onlay	ONL	All partial coverage gold and ceramic restorations including inlays
Cast gold post‐core	GPC	All designs of cast gold posts and posts and cores and gold root caps
Bridges	BRI	All types of bridges irrespective of span or material or abutment tooth preparation, included resin‐bonded bridges, three‐unit and two‐unit bridges
Ceramic Veneer	CVN	All‐ceramic veneers irrespective of preparation design

The differences between the total numbers of unit types were analyzed for significance using two‐tailed paired *t*‐tests with the level of significance set at *p* = 0.05 (GraphPad, Dotmatics). Trend analysis of the total numbers and percentages of different types of laboratory fabricated fixed prosthodontics clinical units was performed using the (two‐tailed) Mann−Kendall trend test (XLSTAT software, Addinsoft). An increasing trend was indicated by a positive S value whereas a decreasing trend was indicated by a negative S value.

## 3 RESULTS

**Figure 1:**
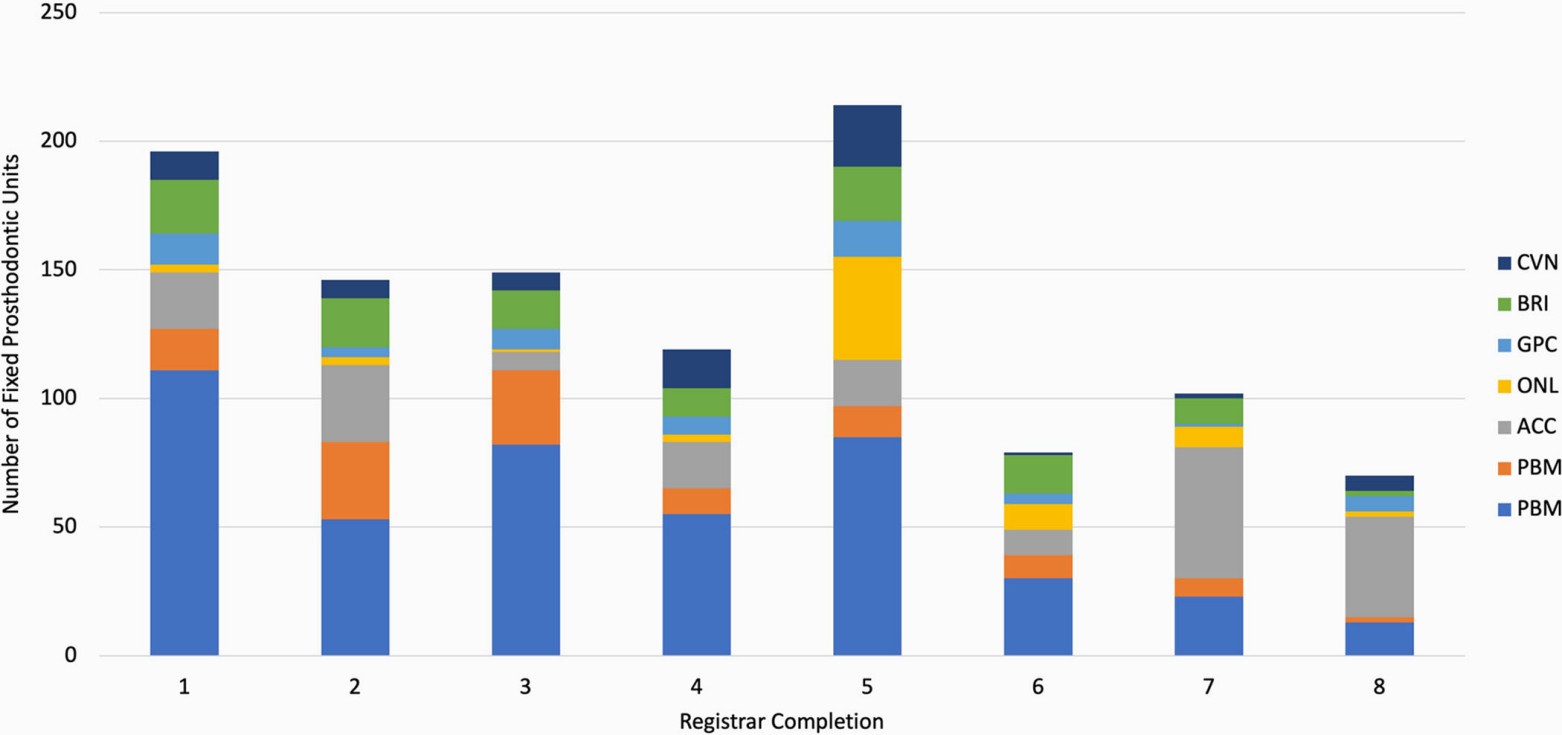
The different types and total numbers of fixed prosthodontics units for each program completion. ACC, all‐ceramic crowns; FGC, full gold crowns; PBM, porcelain bonded to metal.

A total of 1075 laboratory fabricated fixed prosthodontics units were completed in the 8‐year study period representing an average of 134 units per program completion (range 70−214). The final three completions were affected by COVID‐related clinic session closures and restrictions with the average pre‐COVID being 150 units per completion. The different types and total numbers of fixed prosthodontics units for each completion is illustrated in Figure 1.

PBM crowns were most frequently completed representing 42.05% (*N* = 8) of all fixed prosthodontics units over all study years followed by all‐ceramic crown (ACC) (18.14%, *N* = 8) and full gold crown (FGC) (10.70%, *N* = 8). Jointly, PBM, ACC and FGC's encompassed 70.88% (*N* = 8) of the fixed prosthodontics units completed during the study period. PBM crowns were the most used crown in all except for the final two completions where ACC's dominated. The numbers of PBM crowns constructed during each completion were statistically significantly different to all other unit types (two‐tailed paired *t*‐tests, *p* < 0.05) except ACC. Apart from ACC ‐ GPC and GPC ‐ BRI, all other paired restoration analyses using *t*‐tests were not statistically significantly different.

**Figure 2:**
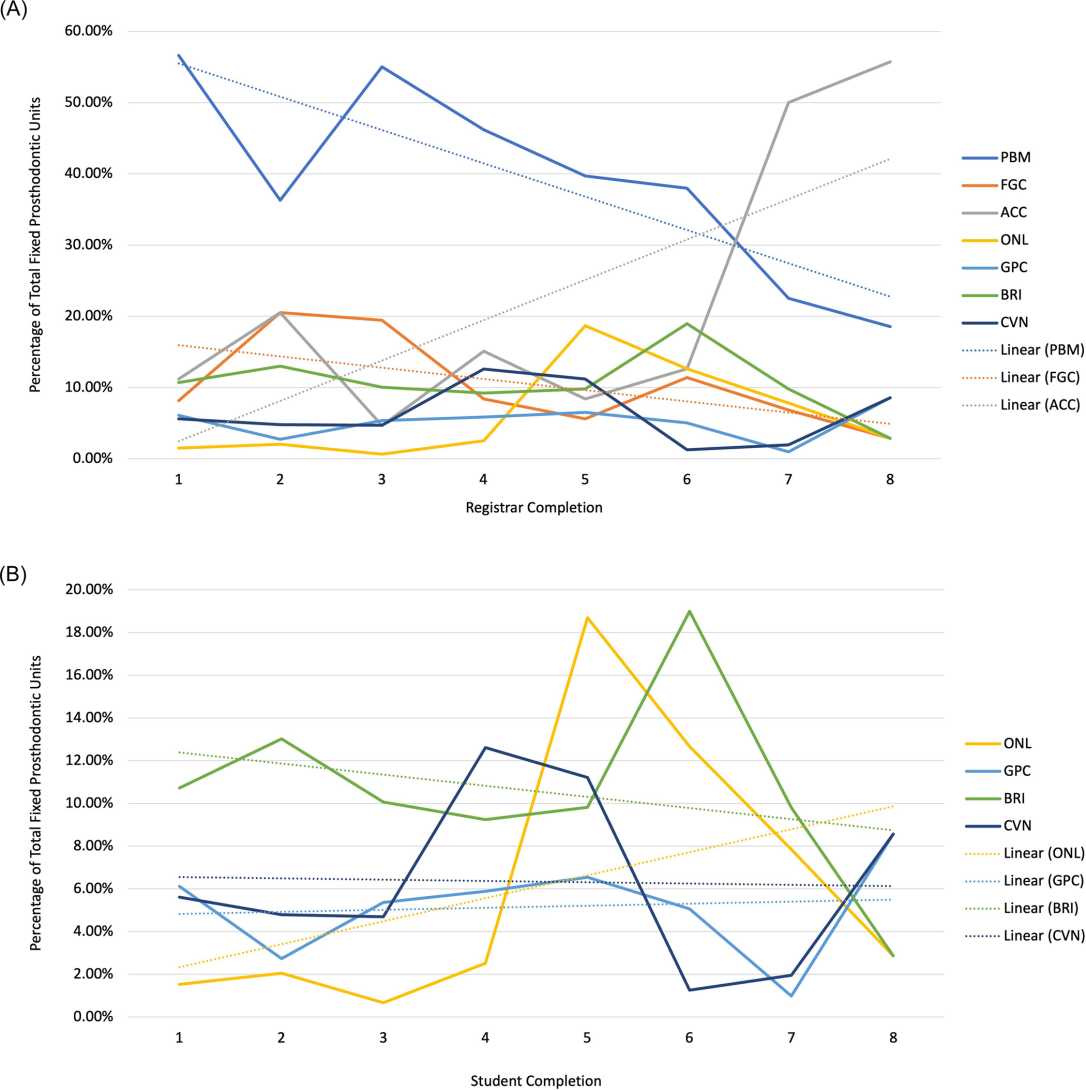
(A) The types of fixed prosthodontics restorations expressed as a percentage of the total units for each program completion for all units with linear trend lines for the dominant values. (B) The types of fixed prosthodontics restorations expressed as a percentage of the total units for each program completion for the minority units with linear trend lines for the minority values.

Figures 2A,B present the types of fixed prosthodontics restorations as a percentage of the total units for each program completion with linear trend lines. The results of the Mann‐Kendall trend tests are provided in Table 2. Although there was a clear visual trend of reduced use of PBM's and increased use of ACC's (Figure 2A), this was not statistically significantly different (*p* > 0.05) using the Mann‐Kendall trend test primarily due to the smaller unit numbers in the final three completions. There was a statistically significant reduction in the use of FGC's (*p* = 0.035). There was a subtle trend of increased use of ONL and reduced use of BRI over the study period that were both not statistically significant (*p* > 0.05) (Figure 2B). The use of CVN and GPC remained relatively constant.

**Table 2:** Mann−Kendall trend analysis of increased or decreased use of fixed prosthodontics unit over the study period.

	PBM	FGC	ACC	ONL	GPC	BRI	CVN
Kendall's tau‐b	−0.619	−0.714	0.429	0.333	0.238	−0.429	−0.143
S value	−13.00	−15.00	9.000	7.000	5.000	−9.000	−3.000
p value	0.072	0.035	0.230	0.368	0.548	0.230	0.764
Significant Trend—Yes/No	No	Yes	No	No	No	No	No

Abbreviations: ACC, all‐ceramic crowns; FGC, full gold crowns; PBM, porcelain bonded to metal.

**Figure 3:**
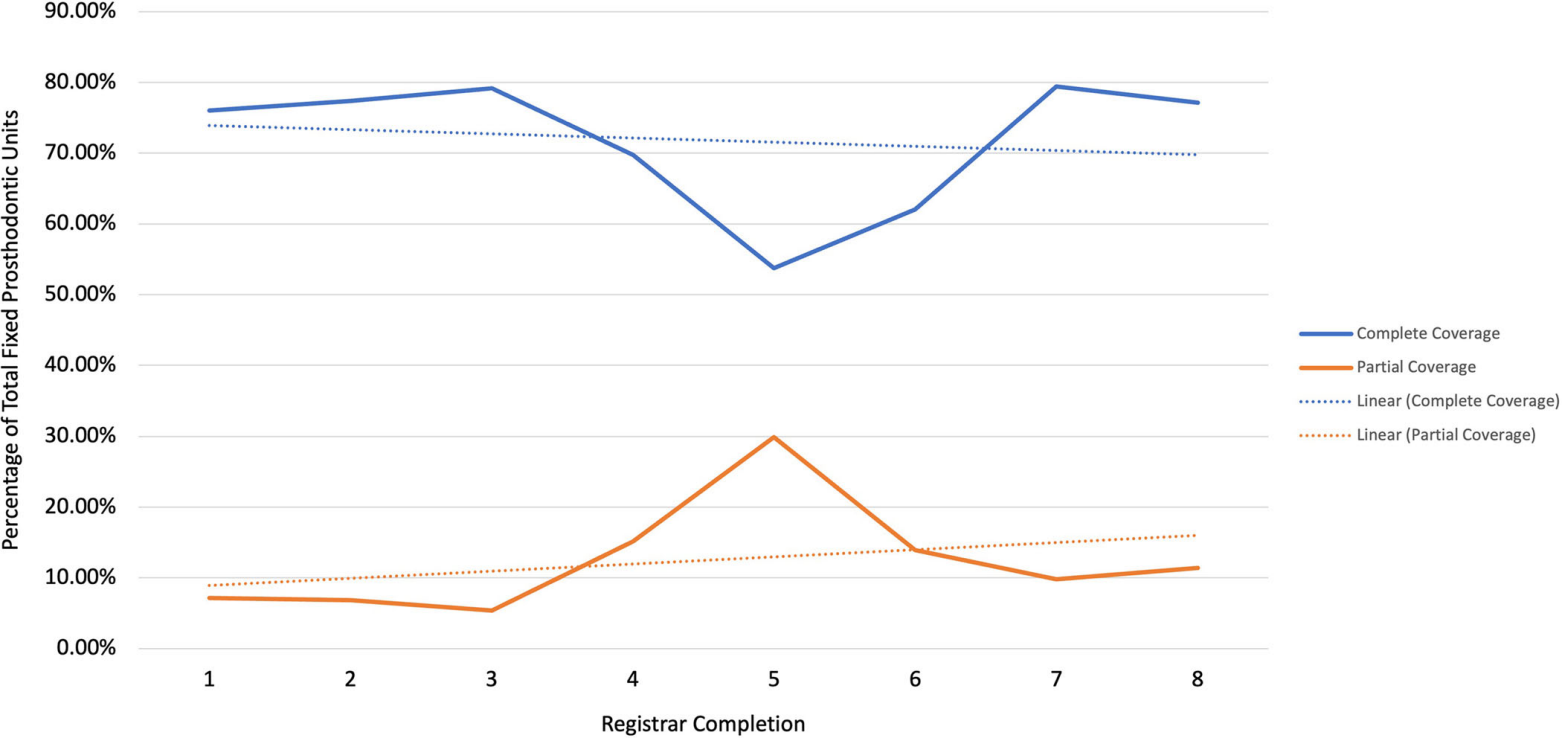
The use of all types of complete coverage restorations (PBM, FGC, and ACC) compared to all types of partial coverage (ONL and CVN) single unit restorations (irrespective of the material and excluding GPC and BRI). ACC, all‐ceramic crowns; FGC, full gold crowns; PBM, porcelain bonded to metal.

There was a statistically significant difference between the use of complete (PBM, FGC, and ACC) and partial (ONL and CVN) coverage restorations (irrespective of the material and excluding GPC and BRI) for each completion (two‐tailed paired *t*‐tests, *p* < 0.001). There was a subtle trend of increased use of partial coverage restorations and reduced use of complete coverage restorations however the change was not statistically significant (*p* > 0.05) (Figure 3). The results were characterized by a sharp downturn of complete coverage and corresponding sharp upturn for completion 5, otherwise the usage remained relatively constant over the study period.

## 4 DISCUSSION

This study embodied pioneer research of its kind and discovered PBM crowns were the most used fixed prosthodontics unit completed over all study years with PBM, ACC and FGC's collectively representing 70.88% of the units. PBM's were the most prevalent crown in all except for the final two completions where ACC's dominated which represented a significant change in practice. There were observed trends of reduced use of PBM's, increased use of ACC's, statistically significant reduced use of FGC's and a statistically significant difference in the use of complete and partial coverage restorations over the study period.

The PBM crown remains the workhorse indirect fixed prosthodontics restoration with high clinical survival rates reported.^12^ In specialist prosthodontics practice, the restoration's versatility is often used to combine aesthetics facilitated by ceramic layering with strength in relatively thin alloy dimensions particularly in cases with limited occlusal space. The increased use of ACC's may be explained by the greater focus on aesthetics by patients and registrars, improved ceramic material properties and the importance on conserving tooth structure that is emphasized at this institution.^13^ There has been a corresponding focus on the use of digital laboratory manufacturing techniques with the purchase of machinery that supports ACC construction.

A comparison to the types of laboratory fabricated fixed prosthodontics clinical units completed by undergraduate students at the same institution^10^ revealed some similar findings with PBM's representing the dominant unit. Collectively, PBM, ACC and FGC's represented 70.88% (present postgraduate study) and 81.80% (undergraduate study) of all fixed prosthodontics units completed. In both studies, there was a trend for reduced use of PBM's and increased use of ACC's over the study period although neither trend reached statistical significance and the trends in the comparative study were less severe. Nevertheless, the trends indicate a significant shift in the clinical practice preferences of fixed prosthodontics at this institution.

The decision to specialize is an important step in a practicing dentist's career. Applicants accept the opportunity cost of financially rewarding private practice to become one of very few prosthodontists and pursue clinical practice of managing very intricate procedures and complex patients. The teaching of the discipline is resource intensive and challenging and requires a deep understanding of the pedagogical principles in a small group discovery experience environment. The context of the present study was limited to analyzing the range of clinical unit experience and associated trends within successful program completions. With an emphasis on quality and reflection rather than achieving set quotas, there were natural variations in individual registrar clinical experiences. On entering the program, the postgraduate prosthodontics registrars were fully qualified and experienced dentists and had at least to some extent developed treatment and material preferences before commencement. Part of the specialist education in prosthodontics is understanding, researching and rationalizing the reasons for material and treatment preferences instead of providing specific answers to specific clinical presentations which does not reflect a case‐based approach in an environment where no one particular selection is necessarily correct. This, in combination with changes in supervising tutors, individual supervising tutor preferences and variations in the treatment decisions according to individual patient presentations may explain some of the unit variations. The final three completions were affected by COVID‐related clinic session closures and restrictions which rationalizes the reduced total unit output.

It was not particularly surprising that the trends of increased partial coverage restorations and decreased complete coverage restorations were not statistically significant due to the nature of the patients presenting at the specialist training clinic. Frequently, patients attend the public sector specialist clinic in a partially dentate state with significant tooth wear and loss of tooth structure and requiring oral reconstruction. The treatment philosophies associated with such presentations are generally not supported by bonded minimal preparation restorations.

This study evaluated the volume of laboratory fabricated fixed prosthodontics clinical units completed which is one measure of performance that lies alongside the more heavily weighted competency‐based prosthodontics assessment.^14^ This is not to say volume of work is not important, however the quality and depth of understanding form the premise of the end of program assessment. Ultimately, teaching institutions aim to produce clinicians who have the technical skills, knowledge, values, personal attributes and professional outlook to deliver highest levels of patient care.^15^ This aligns with the specific educational learning outcomes for this program at this institution that has clinical, didactic and research components and when successfully completed aims to provide graduates with the skill and knowledge required for specialist practice in prosthodontics.

The present study was the first of its kind and conducted over a long time period however there were some limitations. The data was analyzed at a single point in time in a retrospective manner and did not evaluate clinical success. There was a small sample size representative of the number of completions in this specialty at an institution that is one of six in the geographic global region. The reported data is not an all‐inclusive account of all work done in each completion, rather it was limited to laboratory fabricated restorations. There were some fixed prosthodontics treatments excluded due to not involving laboratory fabrication. The logbook format limited the detail in reporting due to its purpose of summarizing information. Some examples included reporting on the restoration location in the mouth, the type of ceramic crown, the type of bridge, and the specific types of alloys used for PBM crowns such as the type of semiprecious alloy. Although there were stringent and consistent recording requirements, there were some natural variations in the detail of information included in the logbooks between individual registrars which limited analysis to the common level of detail. Such as location in the mouth. It is the view of the author that future research should focus on multi‐center reporting of laboratory fabricated fixed prosthodontics clinical units completed in postgraduate prosthodontics specialist training programs. Further research is required to explore the trend indicating replacement of PBM with ACC as the dominant crown type.

## 5 CONCLUSION

PBM crowns were the most used fixed prosthodontics unit completed over all study years with an upsurge in ACC use in the latter study years. PBM, ACC and FGC's collectively represented 70.88% of the units. There were observed trends of reduced use of PBM's, increased use of ACC's, statistically significant reduced use of FGC's and a statistically significant difference in the use of complete and partial coverage restorations over the 8‐year study period.

## AUTHOR CONTRIBUTIONS

**James Dudley**: Conceptualization; data curation; formal analysis; funding acquisition; investigation; methodology; project administration; resources; software; supervision; validation; visualization; writing—original draft; writing—review and editing.

## CONFLICT OF INTEREST STATEMENT

The author declares no conflict of interest.

## ETHICS STATEMENT

Ethics approval was obtained from the Human Research Ethics Committee at The University of Adelaide.

## TRANSPARENCY STATEMENT

The lead author James Dudley affirms that this manuscript is an honest, accurate, and transparent account of the study being reported; that no important aspects of the study have been omitted; and that any discrepancies from the study as planned (and, if relevant, registered) have been explained.

## Data Availability

The data that support the findings of this study are available on request from the corresponding author. The data are not publicly available due to privacy or ethical restrictions.
